# Gene expression profiles of CMS2-epithelial/canonical colorectal cancers are largely driven by DNA copy number gains

**DOI:** 10.1038/s41388-019-0868-5

**Published:** 2019-07-15

**Authors:** Kaja C. G. Berg, Anita Sveen, Maren Høland, Sharmini Alagaratnam, Marianne Berg, Stine A. Danielsen, Arild Nesbakken, Kjetil Søreide, Ragnhild A. Lothe

**Affiliations:** 1 Department of Molecular Oncology, Institute for Cancer Research, Oslo University Hospital, P.O. Box 4953, Nydalen, NO-0424 Oslo Norway; 2 K.G. Jebsen Colorectal Cancer Research Centre, Oslo University Hospital, P.O. Box 4953, Nydalen, NO-0424 Oslo Norway; 3 Institute for Clinical Medicine, Faculty of Medicine, University of Oslo, P.O. Box 4950, Nydalen, NO-0424 Oslo Norway; 4 Department of Gastrointestinal Surgery, Oslo University Hospital, P.O. Box 4950, Nydalen, NO-0424 Oslo Norway; 5 Gastrointestinal Translational Research Unit, Lab for Molecular Biology, Stavanger University Hospital, P.O. Box 8100, NO-4011 Stavanger, Norway; 6 Department of Gastrointestinal Surgery, Stavanger University Hospital, P.O. Box 8100, NO-4011 Stavanger, Norway; 7 Department of Clinical Medicine, University of Bergen, P.O. Box 7804, NO-5020 Bergen, Norway

**Keywords:** Cancer genomics, Colorectal cancer

## Abstract

About 80% of colorectal cancers (CRCs) have chromosomal instability, which is an integral part of aggressive malignancy development, but the importance of specific copy number aberrations (CNAs) in modulating gene expression, particularly within the framework of clinically relevant molecular subtypes, remains mostly elusive. We performed DNA copy number profiling of 257 stage I-IV primary CRCs and integrative gene expression analysis in 151 microsatellite stable (MSS) tumors, focusing on high-level amplifications and the effect of CNAs on the characteristics of the gene expression-based consensus molecular subtypes (CMS). The results were validated in 323 MSS tumors from TCGA. Novel recurrent high-level amplifications (≥15 additional copies) with a major impact on gene expression were found for *TOX3* (16q) at 1.5% frequency, as well as for *CCND2* (12p) and *ANXA11* (10q) at 1% frequency, in addition to the well-known targets *ERBB2* (17q) and *MYC* (8q). Focal amplifications with ≥15 or ≥5 additional copies of at least one of these regions were associated with a poor overall survival among patients with stage I-III MSS CRCs (multivariable hazard ratio ≥3.2, *p* ≤ 0.01). All high-level amplifications were focal and had a more consistent relationship with gene expression than lower amplitude and/or broad-range amplifications, suggesting specific targeting during carcinogenesis. Genome-wide, copy number driven gene expression was enriched for pathways characteristic of the CMS2-epithelial/canonical subtype, including DNA repair and cell cycle progression. Furthermore, 50% of upregulated genes in CMS2-epithelial/canonical MSS CRCs were driven by CNAs, an enrichment compared with the other CMS groups, and associated with the stronger correspondence between CNAs and gene expression in malignant epithelial cells than in the cells of the tumor microenvironment (fibroblasts, endothelial cells, leukocytes). In conclusion, we identify novel recurrent amplifications with impact on gene expression in CRC and provide the first evidence that CMS2 may have a stronger copy-number related genetic basis than subtypes more heavily influenced by gene expression signals from the tumor microenvironment.

## Introduction

Colorectal cancers (CRC) can be classified into four biologically distinct and clinically relevant consensus molecular subtypes (CMS) based on their global gene expression patterns [1]. A few genetic associations to the specific CMS groups have been reported, such as microsatellite instability (MSI) and *BRAF* mutation in CMS1 and *KRAS* mutation in CMS3, and the low frequency of somatic copy number aberrations (CNAs) in MSI is well established. However, little is in general known about the genetic basis for the distinct gene expression-based subtypes.

Chromosomal instability is an integral part of canonical CRC pathogenesis, and the majority of CRCs are characterized by a large burden of CNAs. Chromosomal instability is associated with poor patient prognosis [2] and resistance to multiple drugs [3]. Although the frequencies of individual CNAs in CRC are well studied [4], it remains challenging to distinguish CNAs with impact on tumor growth from passenger events that accumulate as a consequence of chromosomal instability. Although the chromosome instability phenotype is clearly distinct from the MSI phenotype, which is characterized by frequent single-nucleotide variants and small insertions and deletions, there is a large extent of molecular heterogeneity among chromosomally instable tumors.

Known recurrent amplification events are few and of low prevalence in CRC, but may have major impact on precision medicine. The HER2 protein, which is upregulated as a result of high-level *ERBB2* amplification, is a druggable target, and the HERACLES trial demonstrated a 30% response rate to dual HER2 blockade in the 5% subpopulation of HER2 positive and *KRAS* wild-type metastatic cancers [5]. Furthermore, *FGFR2* amplifications are potentially druggable [6, 7], and *KRAS* amplifications may predict lack of response to EGFR blockade [8]. Consistently, the clinical relevance of these amplification events is dependent on subsequent upregulation of the gene product, highlighting the importance of integrative DNA copy number and gene expression analyses.

Gene expression profiles of two of the CMS groups, CMS1-MSI/immune and CMS4-mesenchymal, are heavily influenced by signals from non-malignant cells in the tumor microenvironment, while the profiles of CMS2-epithelial/canonical and CMS3-epithelial/metabolic are largely shaped by cancer cell-intrinsic signals. Consequently, CNAs may have larger effects on gene expression profiles in CMS2 and CMS3.

Here we integrate DNA copy number and gene expression data in primary CRCs to i) identify novel recurrent amplifications with a strong impact on gene expression, and ii) reveal copy number aberrations associated with the distinct gene expression profiles of the individual CMS groups.

## Results

Consistent with the mutation phenotypes, high-resolution DNA copy number profiles from 257 stage I-IV primary CRCs confirmed that MSS tumors (*n* = 203) had significantly more CNAs and loss of heterozygosity (LOH) than MSI (*n* = 53) or MSS *POLE* (*n* = 1) mutated tumors, and the genome-wide frequency of copy number gains and losses recapitulated findings from previous studies (Supplementary Fig. 1a–d). Target genes of focal gains and losses in MSS tumors were found in 25 and 37 chromosomal regions, respectively (GISTIC; *q* < 0.25; Supplementary Table 1), and included a wide range of cancer-critical genes (Cosmic Cancer Gene Census), such as gain of *ERBB2*, *FGFR1*, *FLT3*, *CCND2*, *ERC1* and *CDX2*, and loss of *SMAD4*, *FAS*, *PTEN* and *APC*.

### Novel recurrent high-level amplifications in CRC

**Fig. 1:**
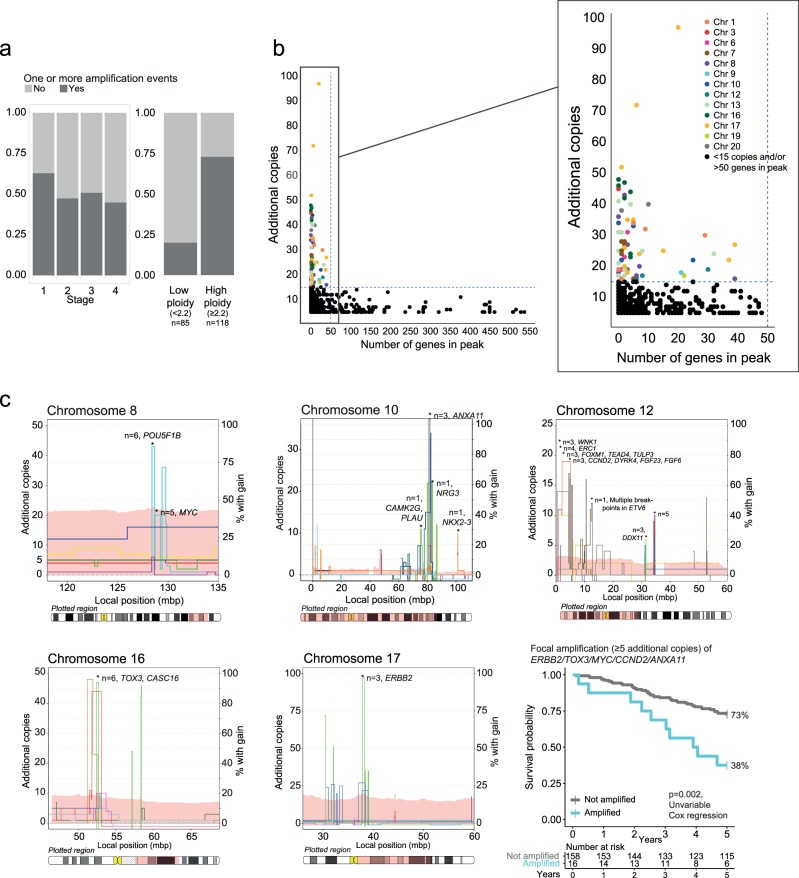
Recurrent high-level amplifications in CRC (**a**) The fraction of tumors with at least one amplification event was not associated with stage, but was substantially higher in high-ploidy tumors compared to low-ploidy tumors. **b** We identified a clear separation between focal and broad amplifications at approximately 15 additional copies and 50 genes in peak. Focal amplifications were distributed across multiple chromosomes (zoomed). **c** On five of the chromosomes with recurrent focal high-level amplifications (≥15 additional copies, <50 genes in peak), additional tumors had focal amplifications with ≥5 additional copies. Including all these amplicons, the amplification frequency was 1.5% (three tumors) for *ANXA11* (10q), *CCND2* (12p) and *ERBB2* (17q); 2.5% (five tumors) for *MYC* (8q); and 3% (six tumors) for *TOX3* (16q). Combined survival analysis showed that the 16 stage I-III MSS patients displaying focal amplifications (with ≥5 additional copies) in either of the five genes demonstrated a significant association to poor patient outcome. Chromosome plots: Left y-axis: number of additional copies above median copy number (different colored lines represent different tumors). Right y-axis: the percentage of samples with gain in the region of interest, visualized as a light pink band behind the lines indicating individual tumors

Approximately half of the MSS tumors (103/203; 51%) had at least one amplification event (median one event per tumor, range 0–25 events; Supplementary Fig. 2a), and this was associated with ploidy, but not with tumor stage (Fig. 1a and Supplementary Fig. 2b). Among high-ploidy tumors (≥2.2n), 74% (86 of 118 tumors) had at least one amplification event, compared with 20% among low-ploidy tumors (17 of 85 tumors with <2.2n).

Amplifications were most frequently seen on 8q, 13q and 20q, which were often affected by broad amplifications (6%, 12% and 14% of amplifications encompassed >200 genes, respectively; Supplementary Fig. 3a–c). A comparison of the number of genes encoded in each amplified peak and the respective amplification amplitude revealed a distinct separation at approximately 50 genes and 15 additional DNA copies, showing that all high-level amplifications (defined as ≥15 additional copies) were focal (encoding ≤ 50 genes; Fig. 1b). Totally, 22 (11%) of the MSS tumors had high-level focal amplifications, but each event was of low frequency. The most prevalent events were recurrent in three tumors each (1.5%) and affected a region on 17q12-q21.1, confirming the existence of a CRC subgroup with extreme *ERBB2* amplification (22, 27 and 97 additional copies), and a region on 16q12.1–12.2 (23, 44 and 47 additional copies), identifying *CASC16* and the transcription factor *TOX3* as novel recurrently amplified genes (Table 1, Fig. 1c, Supplementary Fig. 2e). In addition to the three tumors with high-level amplifications of *TOX3/CASC16*, another three tumors had focal amplifications with 5–14 additional copies of this region, resulting in a total amplification frequency of 3% (≥5 additional copies, 6/203 tumors). Three other high-level amplifications covering *MYC*, *CCND2*, and a region on 10q22.3-q23.1 were recurrent in two samples each (1%). Among stage I-III MSS CRCs, patients with high-level focal amplifications (≥15 additional copies) of either of the five recurrent regions (*n* = 10 patients) had a significantly poorer 5-year overall survival rate than the patients without an amplification (40% versus 71%, *p* *=* 0.03, log rank test). The amplification had independent prognostic value in multivariable analysis including patient age and gender, and tumor localization and stage (hazard ratio [HR] 3.2, 95% confidence interval [CI] 1.3–7.9, *p* *=* 0.01). Similar results were found when including patients with focal amplifications with ≥5 additional copies of these regions in the analyses (total n = 16 patients; Fig. 1c; univariable HR 3.0, 95% CI 1.5–5.9, *p* *=* 0.002, multivariable HR 3.3, 95% CI 1.6–6.8, *p* *=* 0.001; Supplementary Table 2). Of note, neither overall CNA levels, nor the presence of any focal amplification on the ≥5 or ≥15 additional copies levels were associated with poor outcome (*p* > 0.18 in univariable Cox regression). All recurrent amplifications were validated in MSS CRCs from TCGA (Table 1). Furthermore, several cancer-critical genes had high-level amplifications in one tumor each in the inhouse dataset and among these, amplifications of *CDX2* were particularly prevalent in the TCGA cohort, with a 1.6% prevalence of focal high-level amplifications and a prevalence of 4.7% when also including low-level amplifications.

**Table 1:** Amplifications in cancer-critical genes

Cancer-critical genes with focal amplifications^a^	Genomic region	Inhouse cohort, *n* = 203 MSS tumors				TCGA cohort, *n* = 320 MSS tumors			
		≥15 additional copies		≥ 5 additional copies		≥15 additional copies		≥5 additional copies	
		Amplification frequency, % [95% CI]*	CMS type	Amplification frequency, % [95% CI]	CMS type	Amplification frequency, % [95% CI]	CMS type	Amplification frequency, % [95% CI]	CMS type
*ERBB2*	17q12-q21.1	1.5 [0.5–4.3]	CMS2/2/3	1.5 [0.5–4.3]	CMS2/2/3	2.8 [1.5–5.3]	CMS2/2/4/4/4/4/4/NA/NA	2.8 [1.5–5.3]	CMS2/2/4/4/4/4/4/NA/NA
*TOX3 / CASC16*	16q12.1–12.2	1.5 [0.5–4.3]	CMS2/4/NA	3.0 [1.4–6.3]	CMS2/2/2/4/ NA/NA	0.3 [0.02–1.7]	CMS2	0.9 [0.3–2.7]	CMS2/3/4
*MYC*	8q24.21	1.0 [0.3–3.5]	CMS1/2	2.5 [1.1–5.6]	CMS1/1/2/2/ NA	0.3 [0.02–1.7]	CMS3	2.2 [1.1–4.4]	CMS1/2/3/3/4/4/NA
*CCND2 / FGF6 / FGF23 / DYRK4*	12p13.32	1.0 [0.3–3.5]	CMS3/NA	1.5 [0.5–4.3]	CMS3/NA/NA	0.9 [0.3–2.7]	CMS2/2/4	1.9 [0.9–4.0]	CMS2/2/4/4/4/4
*ANXA11* ^b^	10q22.3-q23.1	1.0 [0.3–3.5]	CMS2/NA	1.5 [0.5–4.3]	CMS2/NA/NA	0.3 [0.02–1.7]	CMS4	0.9 [0.3–2.7]	CMS3/4/4
*CDK12/ LASP1* (same peak as *ERBB2*)	17q12-q21.1	0.5 [0.03–2.7]	CMS2	0.5 [0.03–2.7]	CMS2	1.9 [0.9–4.0]	CMS2/2/4/4/4/NA	1.9 [0.9–4.0]	CMS2/2/4/4/4/NA
*CDX2 / FLT3*	13q12.13-q12.3	0.5 [0.03–2.7]	NA	1.0 [0.3–3.5]	CMS2/NA	1.6 [0.7–3.6]	CMS2/2/2/4/4	4.7 [2.9–7.6]	CMS2/2/2/2/2/2/2/3/4/4/4/4/4/4/NA
*ESR1*	6q25.1	0.5 [0.03–2.7]	NA	0.5 [0.03–2.7]	NA	–	–	–	–
*ETV1*	7p21.3–21.2	0.5 [0.03–2.7]	NA	1.0 [0.3–3.5]	NA/NA	–	–	–	–
*FGFR1*	8p11.23–11.22	0.5 [0.03–2.7]	CMS3	2.0 [0.8–5.0]	CMS2/2/2/3	–	–	2.2 [1.1–4.4]	CMS2/2/2/2/2/4/4
*MUTYH*	1p34.1	0.5 [0.03–2.7]	CMS2	0.5 [0.03–2.7]	CMS2	–	–	–	–
*NDRG1*	8q24.22	0.5 [0.03–2.7]	CMS1	2.5 [1.1–5.6]	CMS1/1/2/NA/NA	0.3 [0.02–1.7]	CMS3	0.9 [0.3–2.7]	CMS2/3/4
*RARA / SMARCE1* (same peak as *ERBB2*)	17q12-q21.1	0.5 [0.03–2.7]	CMS2	0.5 [0.03–2.7]	CMS2	0.6 [0.2–2.2]	CMS2/4	1.3 [0.5–3.2]	CMS2/2/4/NA
*SUZ12*	17q11.2	0.5 [0.03–2.7]	CMS3	0.5 [0.03–2.7]	CMS3	–	–	–	–
*SYK*	9q22.2–22.31	0.5 [0.03–2.7]	CMS4	0.5 [0.03–2.7]	CMS4	–	–	–	–
*TOP1*	20q12	0.5 [0.03–2.7]	CMS2	1.0 [0.3–3.5]	CMS2/NA	–	–	2.5 [1.3–4.9]	CMS2/2/2/2/4/4/NA/NA

^a^Focal amplifications were defined as peaks encompassing <50 genes

^b^Not included in the COSMIC cancer census. The gene was nominated based on available literature along with gene expression levels of genes in the amplified peak

^*^CI: 95% binomial confidence intervals using the Wilson method

### Heterogeneity in gene expression concordance of copy number aberrations

Amplification events are expected to have strong effects on gene expression, and 40% of the high-level focal amplification peaks (≥15 additional copies) were associated with outlier expression (defined as >1.5 interquartile range above the third quartile) of at least one affected gene. Moreover, for 76% of the 34 genes with recurrent high-level focal amplifications, the gene expression levels in the affected samples were among the highest in the cohort (see Materials and Methods for criteria). However, including also intermediate-level (≥5 additional copies) and both broad and focal amplification events, no more than 35% (389 of 1096) of recurrently amplified genes had corresponding high gene expression. This indicates a heterogeneous effect of amplifications on gene expression, supporting the expectation that high-level and focal amplifications have the largest impact.

**Fig. 2:**
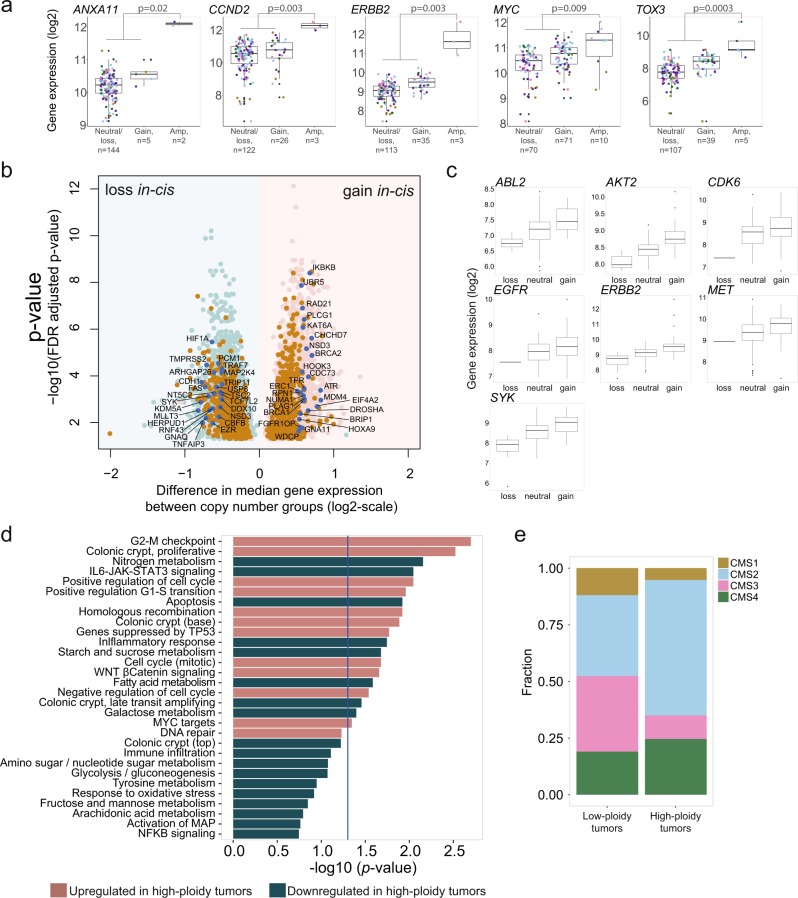
DNA copy number aberrations with concomitant up- or downregulation of gene expression. **a** Gene expression was significantly higher in amplified compared to non-amplified groups in selected recurrently amplified genes. A threshold of 5 additional copies was used as threshold for amplification, and both broad and focal aberrations considered in the 151 tumors where CNA and gene expression data were available. Difference in gene expression was assessed by Wilcoxon rank-sum tests. Colored dots indicate CMS: yellow, CMS1; blue, CMS2; pink, CMS3; green, CMS4; purple, NA. **b** A total of 2467 genes (light pink) were significantly higher expressed in the copy number gain group compared to tumors with neutral copy number state, while 3080 genes (light blue) were significantly lower expressed upon copy number loss compared to tumors with neutral copy number state. All displayed genes were significant with FDR adjusted *p* < 0.05. Orange: genes that were significant in both gain and loss analyses and accordingly had a step-wise increase in gene expression from loss to neutral and gain; dark blue and labeled: cancer-critical genes. **c** Gene expression levels according to CNA status (loss, neutral, gain) for seven oncogenic protein kinases with a significant upregulation of gene expression associated to copy number gain. **d** The high-ploidy group was characterized by downregulation of pathways related to metabolism and immune activation and upregulation of gene signatures related to WNT signaling, cell cycle, homologous recombination, and proliferative colon signature. **e** The high-ploidy group was enriched for CMS2 tumors, shown as the fraction of tumors belonging to each CMS subtype in the low-ploidy and high-ploidy groups. Only CMS classified tumors are shown (*n* = 42 low-ploidy tumors; *n* = 77 high-ploidy tumors)

With respect to the most recurrent amplifications (≥5 additional copies), *ERBB2* amplified tumors had significantly higher expression of both *ERBB2* and other affected genes in this region, and five *TOX3* amplified tumors with available gene expression data had significantly higher expression than non-amplified tumors (Fig. 2a). Amplifications in *CCND2* and *MYC* also had significant impact on gene expression. Furthermore, *ANXA11* was nominated as a likely target of the 10q22.3-q23.1 amplification, based on outlier gene expression in the two analyzed tumors (Fig. 2a). Amplification-associated expression of these genes was also validated in the TCGA tumors, and across both datasets, the increase in gene expression was regardless of CMS subtype (Fig. 2a, Supplementary Fig. 4a).

Genome-wide integration analyses were also performed for recurrent copy number gains and losses (≥10 tumors and with gene expression variance > 0.1). Within the MSS subgroup, 35% of genes with copy number gain (2467 of 7091 genes) and 45% of genes with copy number loss (3080 of 6861 genes) had concomitant upregulated or downregulated gene expression, respectively (false discovery rate, FDR, adjusted *p* < 0.05, Wilcoxon rank-sum test; Spearman’s rho > 0 and FDR adjusted *p* < 0.05, Spearman correlation test; Fig. 2b). In the gain/upregulation category, 4.7% (117 genes) were cancer-critical and 2.1% (53 genes) were classified as oncogenes in MSigDB, among them 15 transcription factors and seven protein kinases (Fig. 2c). Also, 2% (50 genes) were related to DNA-repair, and analyses of pathway overrepresentation (Reactome database) showed a significant enrichment of pathways related to homologous DNA repair and cell cycle when considering genes displaying the largest increase in median gene expression in tumors with gain compared to neutral copy number (Supplementary Table 3). Among genes with loss/downregulation, 4.2% (129 genes) were cancer-critical and 0.8% (25 genes) were classified as tumor suppressors in MSigDB, including *TP53*, *APC*, *SMARCB1* and *SMAD4* and several genes related to differentiation (*CDH1*, *BMPR1A* and *FAS*).

Considering the association between amplification events and a high tumor ploidy, gene set enrichment analyses were also performed in relation to ploidy. This revealed that high-ploidy tumors had upregulation of gene sets related to cell cycle checkpoints, WNT signaling, MYC targets and DNA repair, and downregulation of gene sets related to metabolism, immune response and IL6-JAK-STAT3 signaling (GSA, *p* *<* 0.2*;* Fig. 2d). These processes are CMS-associated, with upregulated gene sets being characteristic of CMS2 and downregulated gene sets of CMS3 and CMS1, indicating a potential link between DNA copy numbers, gene expression and CMS. This was also in line with an observed enrichment of CMS2 tumors in the high-ploidy group (Fig. 2e).

### Frequency variations in DNA copy number and amplification events among the CMSs

**Fig. 3:**
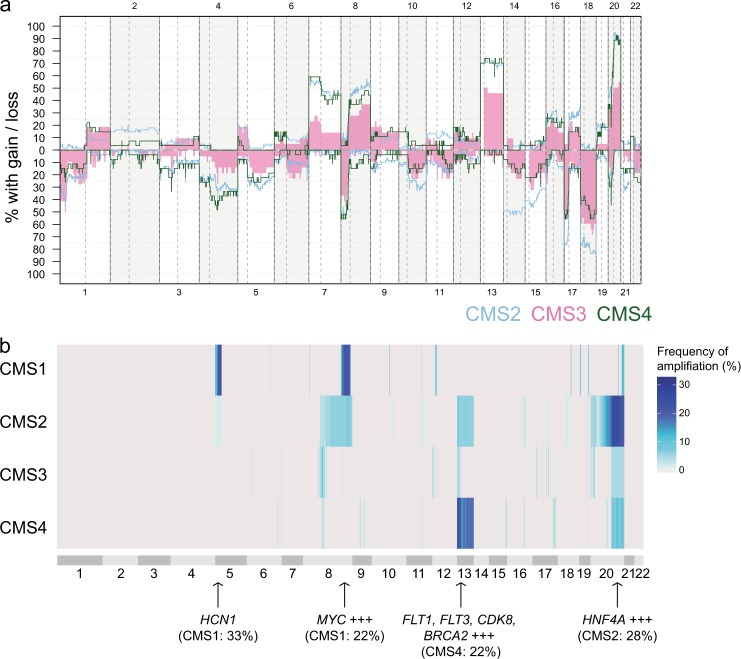
Copy number aberrations relate to CMS (**a**) Frequency of copy number gain and loss in 119 CMS classified MSS tumors, shown according to CMS subtype. CMS1 MSS were excluded from the plot due to low sample size (*n* = 9), gains and losses in CMS1 MSS were included as a separate plot in Supplementary Fig. 1e. Blue: CMS2, pink: CMS3, green: CMS4. **b** The four CMSs differed in the frequencies of amplifications (≥5 additional copies) across the genome (*n* = 119 MSS tumors). The length of the chromosome bands reflects the number of DNA segments and does not directly represent genomic positions. The figure includes data from CMS1 *n* = 9, CMS2 *n* = 61, CMS3 *n* = 22, CMS4 *n* = 27

The genome complexity among MSS tumors, measured as the proportion of bases with aberrant copy numbers, varied (median 24%, range 0–80%) and was associated to CMS (Supplementary Fig. 2c–d). CMS1 MSS had similar levels of genome complexity as CMS2 and CMS4 (median of 35%, 33% and 27% in CMS1/2/4 respectively), but higher levels of LOH than CMS2/4 (median of 31%, 18% and 21%, respectively). In contrast, CMS3 MSS tumors had significantly lower levels of both CNAs and LOH compared to MSS tumors in the remaining subtypes (median 19%, *p* = 0.001 and median 12%, *p* = 0.03, respectively; Supplementary Fig. 2d). No CNAs were exclusive for any of the CMS groups, but there were frequency differences, including a higher frequency of loss on 14q, 17p, and 18p and q in CMS2 compared to CMS4 (>0.25 difference in fraction of tumors with loss) and gain of 10p in CMS4 (18.5% of CMS4 tumors compared to 3.2% of CMS2 tumors; Fig. 3a; Supplementary Table 4 and 5).

With respect to amplifications, CMS1 MSS tumors had frequent events on 5p (33%) and 8q (22%). The 5p amplifications targeted *HCN1* and were specific to this subtype (although the sample number was low, *n* = 9; Fig. 3b). Amplifications on 13q (targeting multiple genes, including *FLT1*, *FLT3* and *CDK8*) and 20q (targeting *HNF4A* and more) were found both in CMS2 and CMS4 tumors, however, 20q amplifications tended to be more frequent in CMS2, while 13q amplifications were more frequent in CMS4 (not statistically significant; 13q: CMS2 8%, CMS4 22%, *p* = 0.09; 20q: CMS2 28%, CMS4: 11%, *p* = 0.1; Fisher’s exact test).

### CMS2-associated gene expression profiles reflect DNA copy number gain

**Fig. 4:**
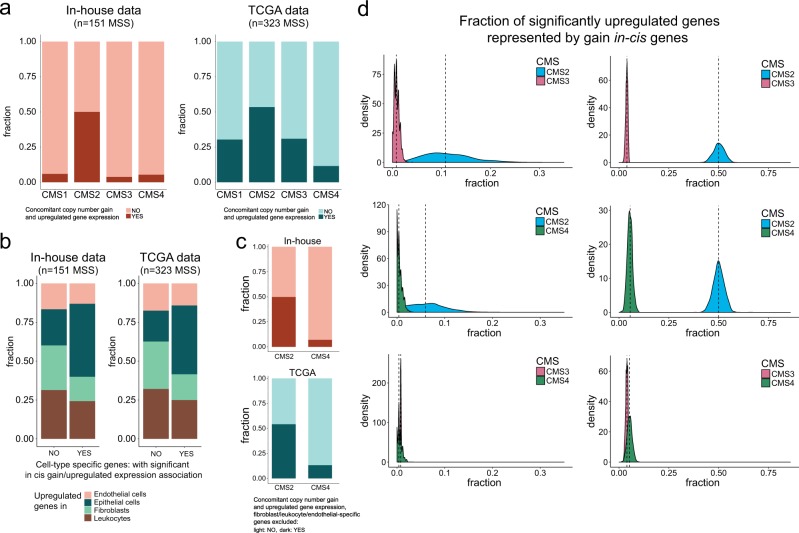
CMS2-associated gene expression profiles are associated to copy number gain. **a** Compared to other subtypes, CMS2 tumors had a substantially larger fraction of significant upregulated genes (limma analysis) that were also associated with copy number gain (in an in cis manner), both in the inhouse cohort and in TCGA data. **b** Analyses of both inhouse and TCGA data revealed that epithelial-specific genes were enriched among genes with significant association between in cis gain and upregulated gene expression, compared to genes preferentially expressed in endothelial cells/leukocytes/fibroblasts. **c** When depleting the list of preferentially expressed genes of fibroblast/endothelial/leukocyte-specific genes, the proportion of genes with significant in cis gain/upregulation remained around 50% in CMS2 and below 15% for CMS4 in both the inhouse and the TCGA cohorts. **d** Left panel: We performed random sampling (*n* = 1000 iterations) of 20 tumors from each subtype with subsequent pairwise calculation of the fraction of CMS-specific upregulated genes consisting of genes in the gain/upregulation category (CMS2 versus CMS3, CMS2 versus CMS4 and CMS3 versus CMS4; CMS1 was excluded due to the low sample number). Specifically, we randomly sampled 250 differentially upregulated genes from the original results on differential gene expression (performed on all CMS classified tumors) and calculated significant gain/upregulated genes separately for each iteration on the 40 sampled tumors. The figure shows the density distribution of fractions calculated for the 1000 iterations. Comparing CMS2 and CMS3, CMS2 had the largest fraction of differentially upregulated genes represented by gain/upregulated genes in all 1000 iterations. Comparing CMS2 and CMS4, CMS2 had the largest fraction of upregulated genes represented by gain in cis genes in 99.4% of the iterations. CMS3 and CMS4 were more similar, with CMS3 having a higher fraction in 64% of the iterations. Right panel: Random repeated sampling of 250 differentially upregulated genes and 250 gain in cis genes (*n* = 1000 iterations) from the original analyses corroborated that CMS2 had a substantially higher fraction of upregulated genes represented by in cis genes compared to other subtypes also when we controlled for the effect of different number of upregulated genes in each CMS group

To explore a potential genetic basis for CMS-associated gene expression profiles in MSS tumors, we investigated the overlap between genes with in cis gain/upregulation and genes that were preferentially expressed in each CMS group (upregulated in comparison with the remaining tumors; limma, FDR adjusted *p* < 0.05 and fold change > 1.2). This showed that CMS2 had a substantially larger fraction of upregulated genes that were also in the gain/upregulation category than the other CMS groups. Among CMS2-upregulated genes, 50% were associated with copy number gain compared to 6%, 4% and 5% in CMS1, CMS3 and CMS4, respectively (Fig. 4a). This was also confirmed in a larger dataset of 323 MSS TCGA tumors, where 30%, 54%, 31% and 12% of preferentially expressed genes were associated with copy number gain in CMS1, CMS2, CMS3 and CMS4, respectively (Fig. 4a). Although the fractions of in cis gain/upregulated genes among preferentially expressed genes in CMS1/CMS3 were higher in TCGA data than in the inhouse data, the substantial differences between the CNA-rich CMS2 and CMS4 subtypes were striking in both analyses. A potential explanation for this is the lower tumor cell fraction of samples in the CMS4 compared to CMS2 subtype (estimated by ASCAT; Supplementary Fig. 4b), reflecting the strong infiltration of particularly fibroblasts in CMS4. Separate analyses of sorted cells from four different compartments of colon cancers (epithelial cells, endothelial cells, fibroblasts and leukocytes; accessed from GSE39396), showed that significant in cis gain/upregulated genes were enriched for genes also upregulated in the malignant epithelial cells (Fig. 4b). To address a potential difference between malignant cells in CMS2 and CMS4, preferentially expressed genes in CMS2 and CMS4 that were also found upregulated in the microenvironment-compartments (fibroblast/endothelial/leukocytes) were excluded from the analysis, retaining 94% and 92% of CMS2-upregulated genes and 39% and 52% of CMS4-upregulated genes for inhouse and TCGA data, respectively. The results from this analysis showed that 50% and 54% of the retained upregulated genes in CMS2 were among the in cis gain/upregulated genes in the inhouse dataset and TCGA, respectively, compared to 7% and 13% in CMS4 (Fig. 4c). Finally, the median correlations between CNAs and gene expression were higher in the CMS2 than CMS4 tumors (Supplementary Fig. 4c). Altogether, these results suggest a stronger copy number-related genetic influence on gene expression in CMS2 than in CMS4, both within the malignant cell compartment and as a result of the strong influence of the tumor microenvironment in CMS4.

To further confirm a significant enrichment of gain/upregulation genes in CMS2 and test that this was not attributable to the larger sample number in this subtype, we performed random samplings (*n* = 1000) of 20 tumors from each CMS group, repeating the in cis gain/upregulation analysis per iteration (CMS1 not included due to the low frequency of CMS1 among MSS samples). The density distributions for the iterations showed that CMS2 had a larger proportion of upregulated genes that were also in the gain/upregulation category than CMS3 and CMS4 in 100% and 99.4% of the iterations, respectively (Fig. 4d; Kolmogorov-Smirnov test for difference in distribution, *p* < 2.2e-16). Additional control analyses by modification of the approach to identify differentially expressed and in cis gain/upregulation genes (outlined in Supplementary Fig. 5) supported the results that CMS2-associated gene expression profiles were specifically enriched for gain/upregulated genes (Supplementary Fig. 5). Additionally, we addressed the potential influence of the variable number of upregulated genes in each subtype by repeated re-sampling of 250 gain/upregulated-category genes and 250 of the preferentially expressed genes in each subtype. The density distributions of the proportion of upregulated genes that were also in the gain/upregulated category across iterations again showed that CMS2 was enriched for copy number-associated gene expression compared with CMS3 and CMS4 (Fig. 4d). The genes that were both upregulated in CMS2 and belonged to the gain/upregulation category were enriched for Reactome pathways related to the cell cycle and DNA repair (homologous recombination and nucleotide excision repair; Supplementary Table 6).

Consistent with the frequent 20q amplifications in CMS2, a total of 11% of upregulated genes in CMS2 were encoded on 20q, compared with 2.4% of upregulated genes in CMS4. In contrast, and although 13q amplifications were more frequent in CMS4, a similar proportion of upregulated genes in CMS2 and CMS4 (4.5 and 2%, respectively) were encoded on 13q, supporting the indication of a more direct effect of copy number gains on gene expression in CMS2 than in CMS4.

## Discussion

Successful targeting of HER2 overexpression has renewed the interest of analyzing high-level amplifications in subgroups of CRC [5]. Still, known amplification events are few and of low prevalence. In this study, by integration of DNA copy number and gene expression data, we conducted a systematic search for amplifications with major impact on gene expression. In addition to the well-known targets *ERBB2* and *MYC*, we identified novel recurrent high-level amplifications of *TOX3* (16q) and *CCND2* (12p) in CRC. The transcription factor *TOX3* was present at more than 23 copies in three tumors in the inhouse dataset, and more than five copies in an additional three tumors, totaling to a 3% amplification frequency in this series of 203 MSS tumors. All *TOX3* amplifications were focal, and the event was associated with increased gene expression, suggesting that *TOX3* may be specifically targeted by amplification in a small subset of CRCs. *TOX3* has been shown to protect neurons from cell death caused by stress originating in the endoplasmic reticulum [9] and to be amplified and overexpressed in breast cancer [10], supporting a cancer-associated function. Furthermore, 1.5% of MSS CRCs displayed focal amplifications (≥10 copies) of *CCND2* with concordant upregulation of gene expression, and D-type cyclins have been shown to confer tolerance to genome-doubling in *TP53* wild-type tumors [11]. Consistently, among the three tumors affected by *CCND2* amplification, one was wild-type for *TP53* and had an estimated ploidy of 4.6, while the two *TP53* mutated cancers had normal ploidy states. Lastly, we propose *ANXA11* as a target for the recurrent (1%) amplification on 10q. The ANXA11 protein has previously been reported to be related to metastasis in CRC [12, 13] and its downregulation has been associated with cell cycle arrest [14]. We confirm that amplification events have a low prevalence in CRC. Although the sample size was not sufficient to analyze prognostic associations for each event individually, combined analyses of *ERBB2*, *MYC*, *TOX3*, *CCND2*, and *ANXA11* indicated that high-level focal amplification of either region was associated with a poor survival among patients with stage I-III MSS CRCs. This was confirmed in the somewhat larger subgroup of patients with focal amplifications at the threshold of ≥5 additional copies of the same regions. Furthermore, all high-level amplifications were focal and had a more consistent relationship with gene expression than broad-range amplifications. Together, this strengthens the role for this type of genomic aberrations in the carcinogenesis of MSS CRCs, and investigation of the clinical relevance of the novel recurrent events in a larger patient series is warranted.

Genome-wide, the effect of recurrent DNA copy number gains and losses on gene expression was heterogeneous, with a concordant in cis-relationship in less than half of the genes with CNAs, which is in accordance with a previous study [15]. By analyzing the data within the CMS-framework we found that among MSS tumors, the gene expression in CMS2 CRCs were driven by CNAs to a much larger extent than the other subtypes, measured as the proportion of upregulated genes with a significant correspondence between gene expression and copy number gain. This was validated in the independent TCGA dataset, which suggested that the difference was most striking between CMS2 and CMS4, despite the similar CNA-burdens in the two subtypes. We propose that this difference is caused by a combination of a stronger infiltration of non-malignant cells in the tumor microenvironment in CMS4 tumors, and a stronger correspondence in DNA copy number/gene expression in malignant cells in CMS2 compared to CMS4 tumors. Statistical re-sampling analyses were performed to rule out the potential confounding effects of varying numbers of samples and upregulated genes in each subtype. This finding was consistent with our hypothesis that the subtypes primarily defined by cancer cell-intrinsic characteristics (CMS2/3) are more directly copy number-driven than subtypes that to a larger extent are shaped by gene expression signals from the tumor microenvironment (CMS1/4). While these analyses do not directly explain why CMS2-related gene expression patterns are more closely associated with CNAs than CMS3-related gene expression, gene set analyses supported that the cell cycle activity characteristic of CMS2 is at least partly explained by DNA copy numbers, and the generally lower CNA burden in CMS3 may be a part of the explanation. Additional functional analyses are needed to confirm that CMS2 tumors are indeed specifically driven by CNAs, and there are limitations to our approach. For example, it has previously been shown that integration of DNA and RNA-level data favors the identification of genes that are more strongly regulated by CNAs than other mechanisms [16], indicating technical bias in the analyses. However, and despite the similar frequencies of CNAs and amplifications in CMS2 and CMS4, our findings may also strengthen the hypothesis that mechanisms other than CNAs have greater importance when it comes to modulating gene expression in the mesenchymal CMS4 subtype. Consequently, we propose that investigation of the functional and clinical consequence of CNAs in larger patient series may benefit from consideration of the transcriptional phenotype within which they occur.

In conclusion, we identify several recurrent high-level amplifications with a major impact on gene expression in MSS CRC, including the known and targetable *ERBB2*, but also novel amplicons such as *TOX3* and *ANXA11*. We also propose that the epithelial/canonical subtype CMS2 has a stronger copy number-related genetic basis than subtypes more heavily influenced by gene expression signals from the tumor microenvironment.

## Materials and methods

### Material

A total of 265 stage I-IV primary CRCs were collected at Oslo University Hospital – Aker (OUH series) in the period of December 2005 to March 2009 and Stavanger University Hospital (SUH series) in the period of June 2003 to November 2009. The OUH series comprised 201 tumor samples from a consecutive study. The SUH series comprised 64 tumors and was intentionally enriched for MSI + tumors (Supplementary Table 2). Principal component analysis of copy number data indicated no systematic differences between the series, and they were consequently analyzed together (Supplementary Fig. 6a).

Of totally 265 collected samples, 257 samples (53 MSI, 1 MSS *POLE* mutated and 203 MSS samples) were included for downstream analyses (samples with failed ASCAT segmentation, *n* = 2; low tumor percent, *n* = 2; and inconclusive MSI analyses, *n* = 4 were excluded). Gene expression data was available for all CRCs in the OUH series that were not treated with preoperative chemoradiotherapy (*n* = 189). Pre-operative treatment did not have a major impact on the CNA data (assessed by principal component analysis, PCA; Supplementary Fig. 6a).

The study was approved by the Regional Committee for Medical and Health Research Ethics, South East Norway (project ID: 1.2005.1629) and the Regional Committee for Medical and Health Research Ethics, West Norway (project ID: 197.04). All patients provided written informed consent, and the study was conducted in accordance with the Declaration of Helsinki.

### DNA and RNA extraction and MSI analysis

DNA and RNA were extracted using the Qiagen AllPrep DNA/RNA Mini kit (Qiagen, Hilden, Germany) according to manufactures recommendations. Briefly, tissue was homogenized and lysed using the Qiagen TissueRuptor and DNA and RNA was extracted using spin columns. One extra cycle of washing was performed for removal of residual buffer before elution of DNA and concentration and ratios were measured with Nanodrop (Thermo Fisher, Waltham, MA, USA). MSI status was determined by analyses of the BAT-25/BAT-26 mononucleotide loci, according to procedures previously described [17]. Seven samples had uncertain MSI status, two of which were determined by additional analyses using the MSI Analysis System, version 1.2 (Promega, Fitchburg, WI, USA), and one sample was grouped as an MSS due to its complex copy number profile. The remaining four samples were excluded from group analyses. Analyses of *POLE* mutation status has previously been performed for the OUH samples [18], and one POLE mutated MSS tumor was grouped with MSI tumors for all subgroup analyses.

### DNA copy number analysis

Copy number data were generated using Affymetrix Genome-Wide SNP6.0 arrays according to recommended procedures and as previously described [19]. In short, 500 ng of DNA in low-EDTA TE-buffer was digested, adapter ligated, diluted, amplified and quality controlled by gel electrophoresis (4% TBE gel, Lonza, Basel, Switzerland). Samples were purified using AMPure XP beads (Beckman Coulter, Brea, CA, USA) and further fragmented and quality controlled by gel electrophoresis (2% TBE gel, Lonza) prior to labelling, denaturation and hybridization onto arrays for 16–18 h. Hybridized samples were washed, stained and scanned, and quality cut-offs of CQC > 0.4 and MAPD < 0.34 were set for inclusion of samples into the final dataset. Resulting CEL files were pre-processed using PennCNV-Affy using Affymetrix Power Tools version 1.15.0 as previously described [20].

Segmentation of individual samples was performed by two methods: i) by the PCF algorithm implemented in the R package copynumber (version 1.16.0 and R version 3.4.0) [21] with gamma parameter 100, and ii) by the ASCAT algorithm (version 2.4.4 and R version 3.3.2) [22] with penalty parameter 50. Relative copy numbers (mean LogR ratios) from PCF were used to calculate the percentage of genome affected by gain and loss, for genome wide gain/loss frequencies across sample groups, for GISTIC analysis, and for integration of copy number gain/loss and gene expression data). Allele-specific ASCAT data (whole gene copies) were used to analyze amplifications and LOH, and for estimation of ploidy. Chromosomes X and Y were disregarded from analyses.

The PCF data was median centered within each sample prior to downstream analyses, and PCF values of 0.15/-0.15 were used as threshold for calling copy number gain and loss. All downstream analyses were run using R version 3.4.1.

The fraction of the genome with aberrant copy number/LOH was calculated as the number of aberrant bases out of the total number of bases with copy number/LOH estimate available. The PCF and ASCAT data had good concordance in estimation of the fraction of the genome affected by gain/loss among the samples (Supplementary Fig. 6b). Amplifications were called from ASCAT data as total copy numbers (nAB) more than five copies above the sample-wise genome-wide median copy number as estimated by ASCAT. LOH was defined when nA or nB was zero and the remaining allele was non-zero.

GISTIC analysis (version 2.0.23) was performed using default parameters, except for ta/td 0.15, brlen 0.7, maxseg 2000, conf 0.99, genegistic 1, broad 1, and savegene 1. Significant regions based on an FDR adjusted p-value (i.e., *q*-value) threshold of 0.25 were reported.

### Gene expression analysis

Gene expression data obtained using exon-level Affymetrix GeneChip Human Exon 1.0 ST arrays and pre-processed according to RMA, have previously been published for the OUH series (GSE24550, GSE29638, GSE69182) [23]. Transcript clusters with missing gene symbols were excluded, and genes with annotations from different databases were reduced to one entry per gene by prioritizing according to RefSeq, ENSEMBL, UCSC genes, Genbank and GenbankHTC. Gene symbols were checked by the multi-symbol checker available from the HUGO Gene Nomenclature Committee (HGNC) to ensure that the current gene symbols were used, for proper alignment with the CNA dataset [24]. Differential gene expression analysis was performed with the R package limma (version 3.32.10). Differences in gene set scores between groups for 66 gene sets (MSigDB v6.1) relevant for CRC or chromosomal instability were assessed by the R package GSA (version 1.03; Supplementary Table 7) and gene sets with *p*-values < 0.2 were reported. Reactome pathway enrichment analysis was done using the PANTHER Overrepresentation test in the online PANTHER tool [25] with Reactome version 58 [26].

The samples have previously been classified according to CMS using the classifyCMS.RF function in the R package CMSclassifier, with default posterior probability threshold [27]. Confident classification was obtained for 149 samples in the OUH series, 119 of which were MSS (Supplementary Fig. 7a).

### Integration of gene expression and DNA copy number data

Integration of DNA copy number and gene expression data was performed for 151 MSS from the OUH series (Supplementary Fig. 7b). Genes were assigned copy number values from PCF segmented data by mapping hg19 genome coordinates to transcripts as annotated in the R package TxDb.Hsapiens.UCSC.hg19.knownGene (version 3.2.2) using the findOverlaps function from the GenomicRanges package (version 1.28.4). Genes with conflicting copy number values along the gene, i.e. spanning segment breakpoints were handled as follows: (i) genes where all segments were called with either loss or gain kept the most extreme copy number estimate, (ii) genes where all segments were copy number neutral were assigned the median PCF value of those segments, (iii) genes that ranged between neutral copy number and gain or loss were called as aberrant (kept the most extreme value), and (iv) genes with both gain and loss were set to NA for that particular sample. Prior to integration analyses, genes with expression variance of <0.1 across samples were excluded. Gene expression was compared between samples in different copy number groups (copy number gain versus neutral copy number, or loss versus neutral copy number) using Wilcoxon rank-sum tests (two-sided) and a significance threshold of FDR corrected *p* < 0.05 (genes with <10 samples in each copy number group were not considered). The p.adjust function implemented in the R stat package was used for FDR correction. In cis-association between gene expression and copy number was called only for genes that also had a positive correlation between gene expression and copy number from Spearman’s test (Spearman’s rho > 0 and FDR corrected *p* < 0.05).

Correspondence between gene amplification and expression was assessed in two ways. First, for an amplification peak-oriented approach of focal amplifications only, we determined whether at least one gene in each amplified peak had outlier gene expression, defined as expression levels higher than 1.5 times the interquartile range above the third quartile of all 151 MSS tumors. Second, for a gene-focused analysis of both broad and focal amplifications, we investigated the effect of amplification events (≥5 additional copies) found recurrently (≥2 tumors) across the MSS cohort, for genes with gene expression variance of >0.1. Genes were identified as having an in cis-association if at least 50% of the amplified tumors were among the top [1.5*tumors with amplification] samples on gene expression (or among top five samples on gene expression when fewer than five samples displayed amplification). Similar correspondence analyses were also performed for recurrent high-level amplifications, defined as ≥15 additional copies in >1 tumor. For selected amplified genes the gene expression was additionally assessed by Wilcoxon tests (amplified versus non-amplified groups, both broad and focal amplifications of ≥5 additional copies considered as amplified).

To investigate a potential enrichment of genes with in cis-association (gain/upregulation) among upregulated genes in each of the four CMSs we identified differentially expressed genes between each subtype and the remaining subtypes using limma (FDR adjusted *p* < 0.05 and fold change > 1.2) and calculated the proportion of upregulated genes that overlapped with in cis genes. To control for the potential impact of different numbers of samples in the subtypes, we performed random sampling (*n* = 1000) of 20 tumors from each CMS group and repeated analyses for each iteration. Enrichment was assessed in pairwise comparisons among the subtypes. For a gene to be assessed in the in cis analysis, at least three tumors had to be found in the copy number gain and copy number neutral group. For robustness, the analysis was done in three different ways: (i) by identifying significant in cis genes (gain/upregulation, Wilcoxon rank-sum tests) for the 40 tumors (20 tumors in each of the subtypes included in the pairwise comparison) and performing differential expression analysis (limma; FDR adjusted *p* < 0.05 and fold change > 1.2) for the 20 versus 20 tumors per re-sampling, (ii) by identifying significant gain/upregulated genes for the 40 tumors per re-sampling and using the differential expression results from the original analyses including all samples, and (iii) by identifying significant *in cis* genes for the 40 tumors and randomly sampling 250 genes from the original differential expression results. Furthermore, to test if the variable number of genes that were differentially upregulated in each subtype affected the analysis we also performed repeated (*n* = 1000) re-samplings of 250 genes from the original gain/upregulated gene list and among the genes originally found to be upregulated in each CMS group (CMSx versus remaining CMS classified tumors).

### TCGA validation analyses

To validate both the nominated high-level DNA amplifications and CMS-related associations between CNAs and gene expression, 323 MSS tumors from the TCGA colon/rectal tumor dataset with both data types available were analyzed. The CNA data was downloaded as raw CEL files and analyzed with the same analysis pipeline used for analyses of the inhouse data. Matching gene expression data (RSEM-normalized values) were used for integration analyses with CNAs. Prior to data integration, the same fraction of low-variance genes as in the inhouse dataset was excluded prior to Wilcoxon-testing. The amplification analyses (based on allele-specific ASCAT segmented data) were performed on 320 out of 323 tumors due to segmentation failure in 3 samples.

### Contribution of different CRC cell compartments

Gene expression data from four different colon cancer cell populations (epithelial cells, endothelial cells, fibroblasts and leukocytes; GSE39396 [28], isolated from tumors using Fluorescence Activated Cell Sorting) was included in the analyses to investigate the contribution of different cell compartments to the preferentially upregulated genes in the CMS groups and the significant in cis gain/upregulated genes. Differential gene expression analyses were performed using limma, as described above.

### Survival analyses

Survival analyses were performed for patients with stage I-III MSS CRCs (*n* = 174; Supplementary Table 2). Five-year overall survival was used as endpoint, evaluating time from surgery to death from any cause. Patients without an event were censored at 5 years. Kaplan-Meier plots were generated with the ggsurvplot function in the R package survminer. The survdiff function in the R package survival was used for log rank tests. Univariable and multivariable Cox proportional hazards analyses were performed with the coxph function implemented in the R package survival. For multivariable analyses, age (above/below median), gender, tumor localization (distal/proximal) and stage were included as variables.

## Supplementary information

Supplementary Figures

Supplementary Tables (PDF)
