# Supplementary material for: Gene expression profiles of CMS2-epithelial/canonical colorectal cancers are largely driven by DNA copy number gains
Source: Oncogene. 2019 Jul 15;38(33):6109–22. doi: 10.1038/s41388-019-0868-5 (PMC6756070; doi:10.1038/s41388-019-0868-5)
Supplement: Supplementary file 1 — Supplementary Figures [file 41388_2019_868_MOESM1_ESM.pdf]

Supplementary Figure 1

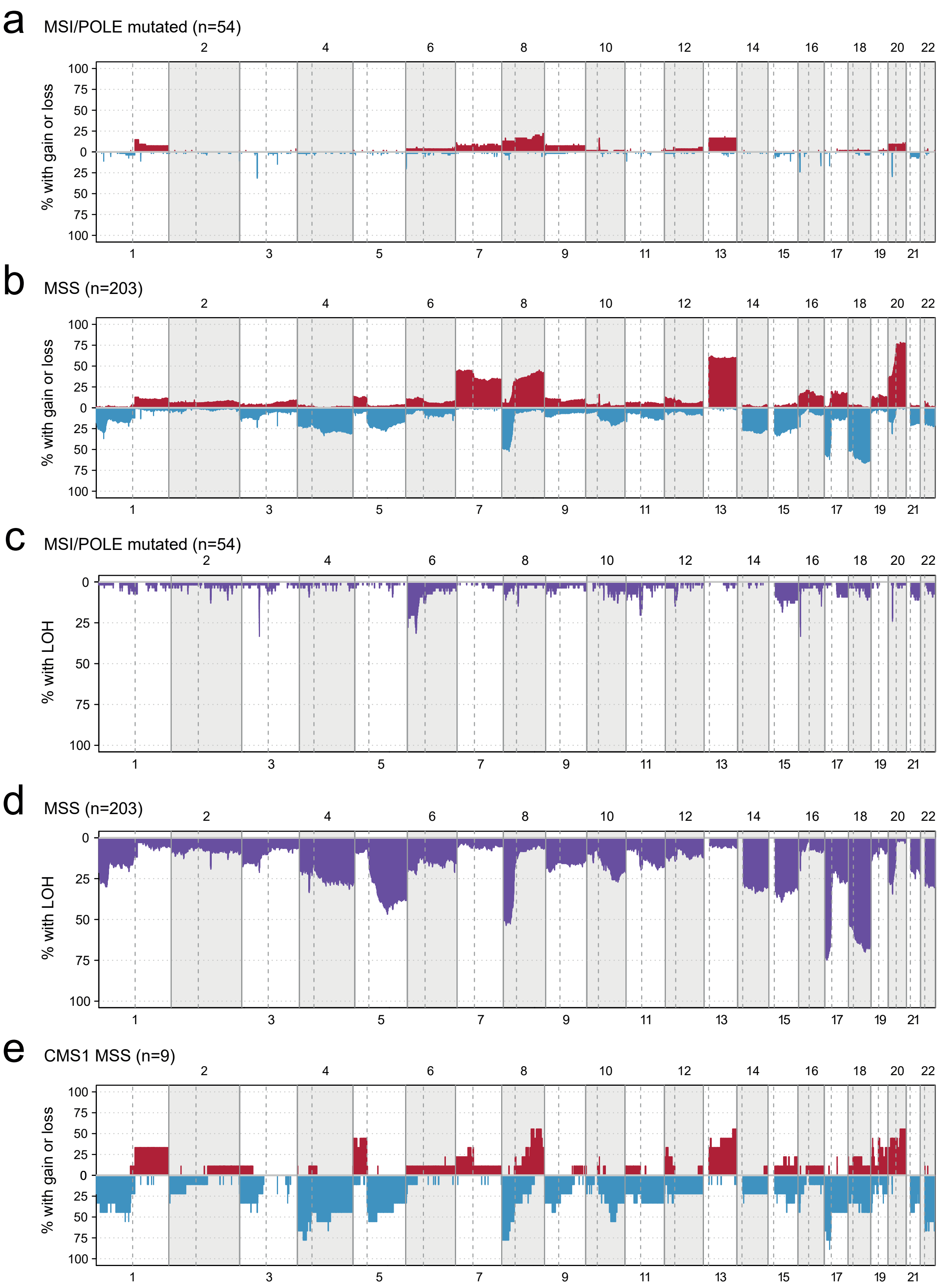

Supplementary Figure 1. a) Frequencies of copy number aberrations in 54 MSI or *POLE* mutated tumors. The most frequent CNAs (>15%) paralleled events found at higher frequencies in MSS tumors, including broad level gains on 8 p and q, 10q and 13q. Copy number gain of 8q24.3 was the most frequent event (12/54 tumors). Additionally, MSI tumors had frequent focal losses, mainly in fragile genomic regions, targeting the genes *FHIT* (3p14.2), *GMDS* (6p25.3), *RBFOX1* (16p13.3), *WWOX* (16q23.1), *LGALS9C* (17p11.2), and *MACROD2* (20p12.1). b) MSS tumors had gain of 7p and q, 8q, 13q and 20p and q (>40% of tumors), and losses covering broad chromosomal regions on 8p, 17p and 18p and q (>40% of samples), all of which are well-known events in MSS CRC. c) Events of LOH in MSI were recurrent on 3p, 6p, 16p and 20p. d) In MSS tumors, 5q, 8p, 17p and 18p and q were most frequently affected by LOH. e) Frequencies of CNAs in CMS1 of the MSS phenotype (n=9).

Supplementary Figure 2

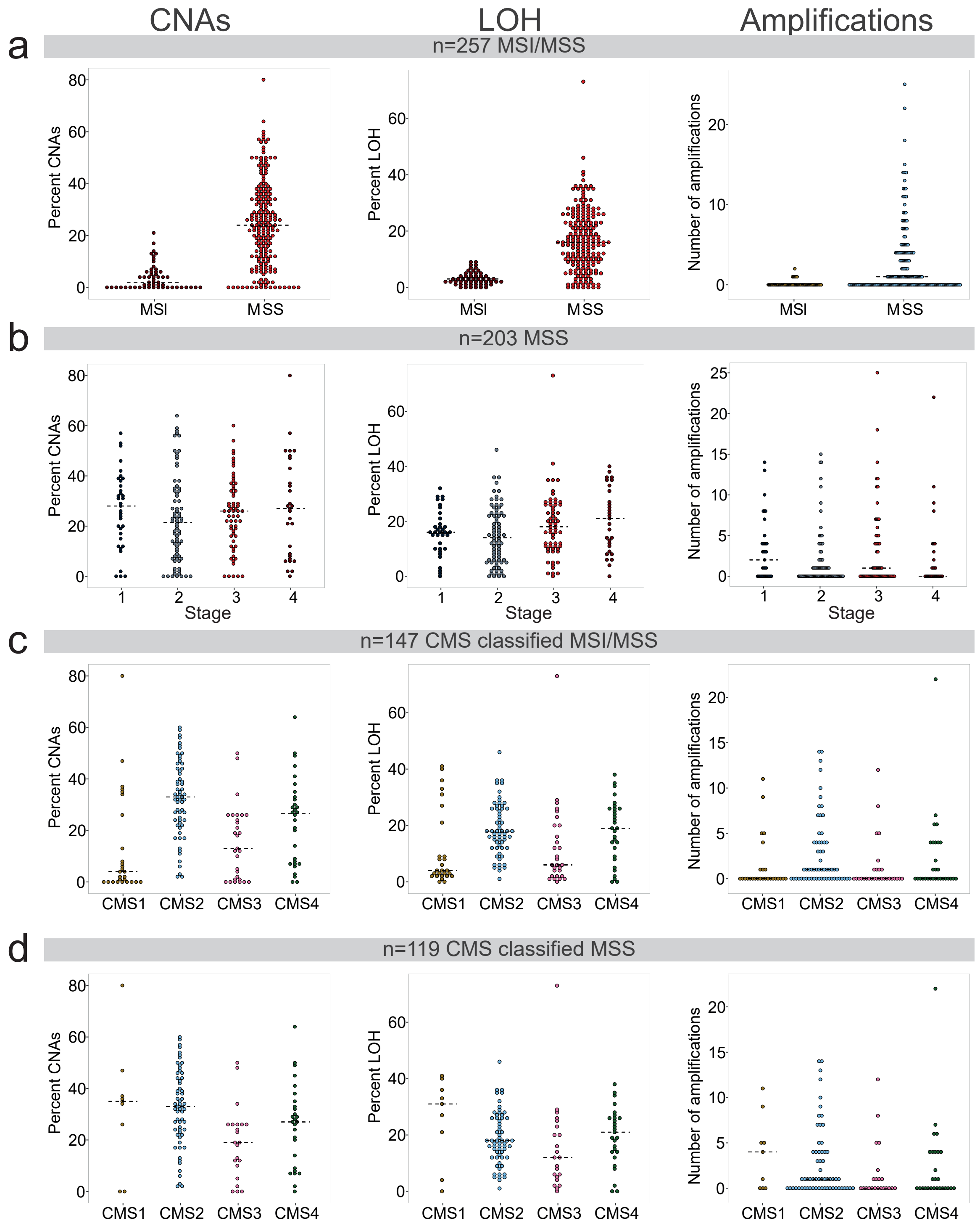

e

	Chrom	Stage	CMS
FGFR1	8	1/1/2/2	CMS2/2/2/3
MYC	8	2/3/3/3/4	CMS1/1/2/2/NA
ANXA11	10	1/3/3	CMS2/NA/NA
CCND2	12	1/2/3	CMS3/NA/NA
ERC1	12	1/2/3/3	CMS3/NA/NA/NA
TOX3/CASC16	16	1/2/2/3/3/4	CMS2/2/2/4/NA/NA
ERBB2	17	1/2/3	CMS2/2/3

Supplementary Figure 2. a) MSI tumors (n=54) had significantly lower CNA levels compared to MSS tumors (n=203), in terms of the percentage of genome with aberrant copy number (left) and LOH (middle) and amplifications (right); one MSS *POLE* mutated tumor was grouped along with MSI for all analyses due a hypermutated phenotype; Wilcoxon rank sum tests, p<0.001). b) Neither CNA levels (left) nor amplifications (right) was associated to tumors stage (stage 1+2 *versus* stage 3+4, p=0.38 and 0.99, respectively; n=203 MSS tumors). This was in contrast to LOH levels, which were higher in stage 3+4 compared to stage 1+2 (middle; p=0.02). c) Considering all CMS classified tumors (n=147), CMS1 displayed lower levels of CNA and LOH compared to remaining subtypes (p<0.001 and p<0.01). The CMS2 subtype had significantly higher levels of both CNAs and LOH (p<0.01) and more amplification events than other subtypes (p<0.001). d) Considering CMS classified MSS tumors only (n=119), CMS1 tumors were comparable to tumors from other subtypes with regards to CNAs (p=0.2), and had slightly higher LOH levels, although the group size was small (n=9; p<0.05). In terms of amplifications, the median number of events in CMS1 tumors were almost 5 events, although the high median seemed to be driven by a few samples with comparable numbers of amplification events as that observed in some CMS2 tumors. e) Overview of stage and CMS class for tumors displaying recurrent focal amplifications (≥5 additional copies) among 203 MSS tumors.

Supplementary Figure 3

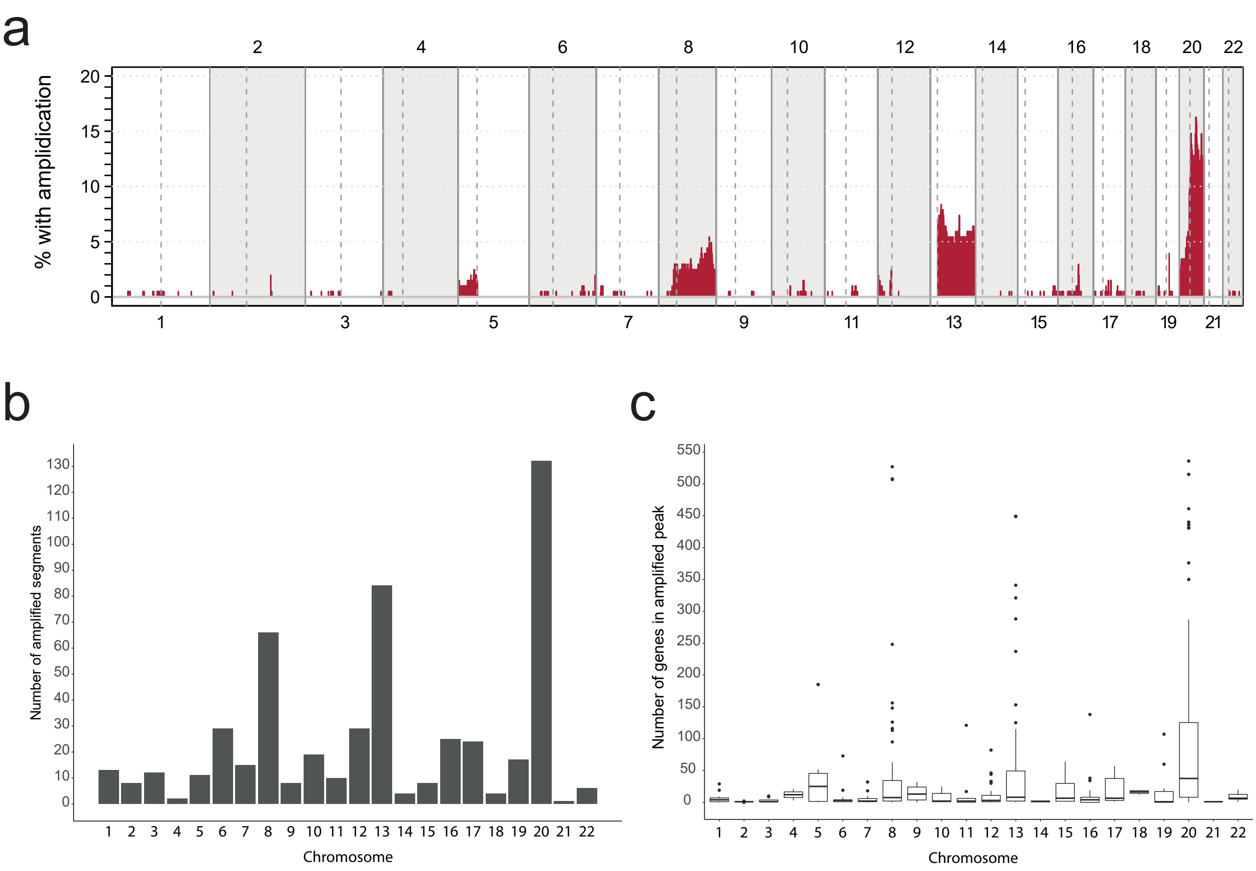

Supplementary Figure 3. a) Genome-wide overview of amplification frequencies, defined as ≥5 additional copies (n=203 MSS). Chromosome arms 8q, 13q and 20q were most frequently affected by amplifications. b) The number of amplification events (y-axis) for chromosomes 1 through 22 (x-axis). c) The number of genes in amplified peak (y-axis) for chromosomes 1 through 22 (x-axis). Chromosome arms 8q, 13q and 20q were the only chromosome arms affected by amplification events encompassing >200 genes in the amplified peak.

Supplementary Figure 4

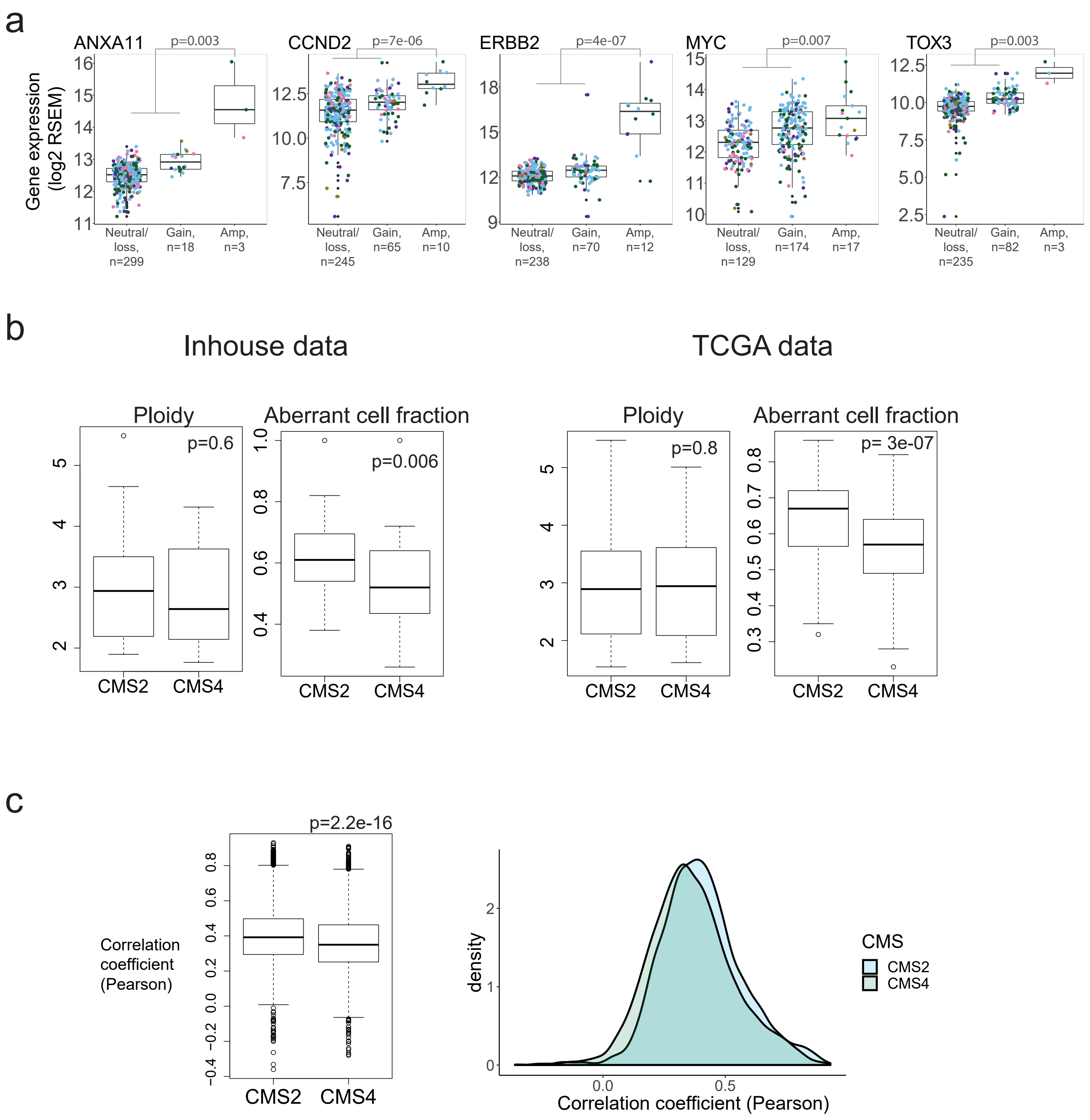

Supplementary Figure 4. a) Validation analyses in a TCGA cohort of 320 MSS tumors confirmed that the nominated amplicons from inhouse data anlysis also were found amplified in TCGA data, with a significant impact on gene expression in the affected tumors. b) CMS2 tumors had higher aberrant cell fractions, as estimated by the ASCAT algorithm, compared to CMS4 tumors, both in 151 tumors from an in-house dataset and in 320 TCGA tumors. Ploidy levels in the two CMS groups were similar. c) Among 4341 genes with significant correspondence between copy number gain and gene expression (nominated from analyzes on all 323 tumors in the TCGA dataset), the Pearson correlation between copy number state and gene expression was signficantly higher in CMS2 tumors compared to CMS4 tumors, assessed by a t-test.

Supplementary Figure 5

Alternative analysis A:
Differential expression analysis
and *in cis* analysis per iteration

Alternative analysis B:
Original differential expression analysis
In cis analysis per iteration

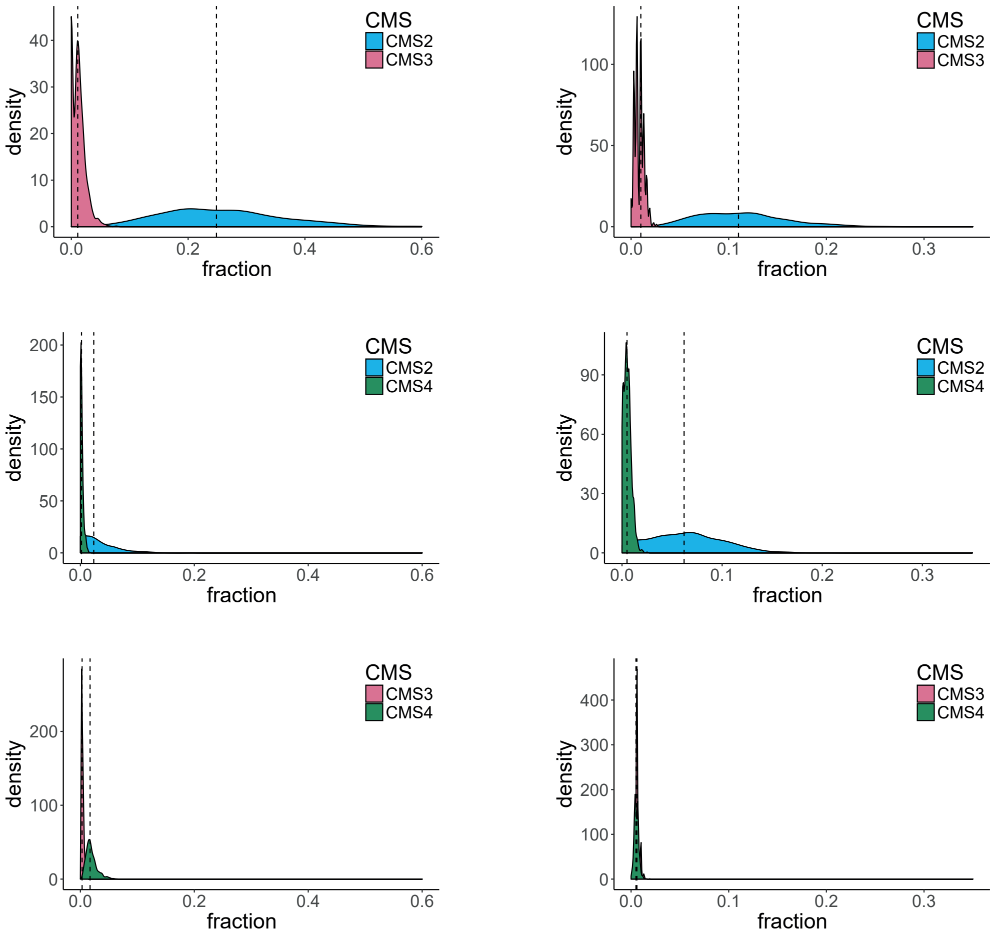

Supplementary Figure 5. Repeated re-sampling analysis to confirm enrichment of in cis (gain/upregulated) genes in CMS2. In addition to the results shown in Figure 4, two alternative methods for repeated re-sampling was performed. Left panel (alternative analysis A): The differential gene expression analysis and the *in cis* analysis was both performed separately per iteration on the 40 randomly sampled tumors (20 tumors from each subtype). The differential gene expression analysis was hence performed on 20 versus 20 tumors in each iteration. The *in cis* analysis was performed on the 40 tumors combined. For each iteration the fraction of differentially upregulated genes that were also found to be significant *in cis* genes was calculated, producing a density of fractions depicted in the figure. This analysis was hence more independent of the group size, but also had limitations: the low sample number prevented the approach of performing differential gene expression analysis on CMSx versus remaining CMSs, as was done in the original differential gene expression analysis. Consequently, the differential gene expression analysis was performed by comparing CMSx versus CMSy. Also, the number of upregulated genes in this analysis were more systematically different between groups (for instance CMS4 had much more upregulated genes than CMS2 and CMS3 in their pairwise analyses). Regardless of these limitations, the results showed that in 100% and 88% of the cases, CMS2 had a higher fraction of upregulated genes represented by *in cis* (gain/upregulated) genes as compared to CMS3 and CMS4 respectively. Right panel (alternative analysis B): For each iteration, the results from the original differential gene expression analysis were used to identify upregulated genes in each CMS, while the *in cis* analysis was performed on the 40 randomly sampled tumors. For every iteration, the fraction of originally upregulated genes found among significant *in cis* genes from that iteration was calculated, and the plot shows the resulting density of fractions. Also here, CMS2 had a higher fraction of upregulated genes represented by *in cis* genes in 100% of the iterations in comparison with CMS3 and 99.9% of the iterations in comparison to CMS4.

Supplementary Figure 6

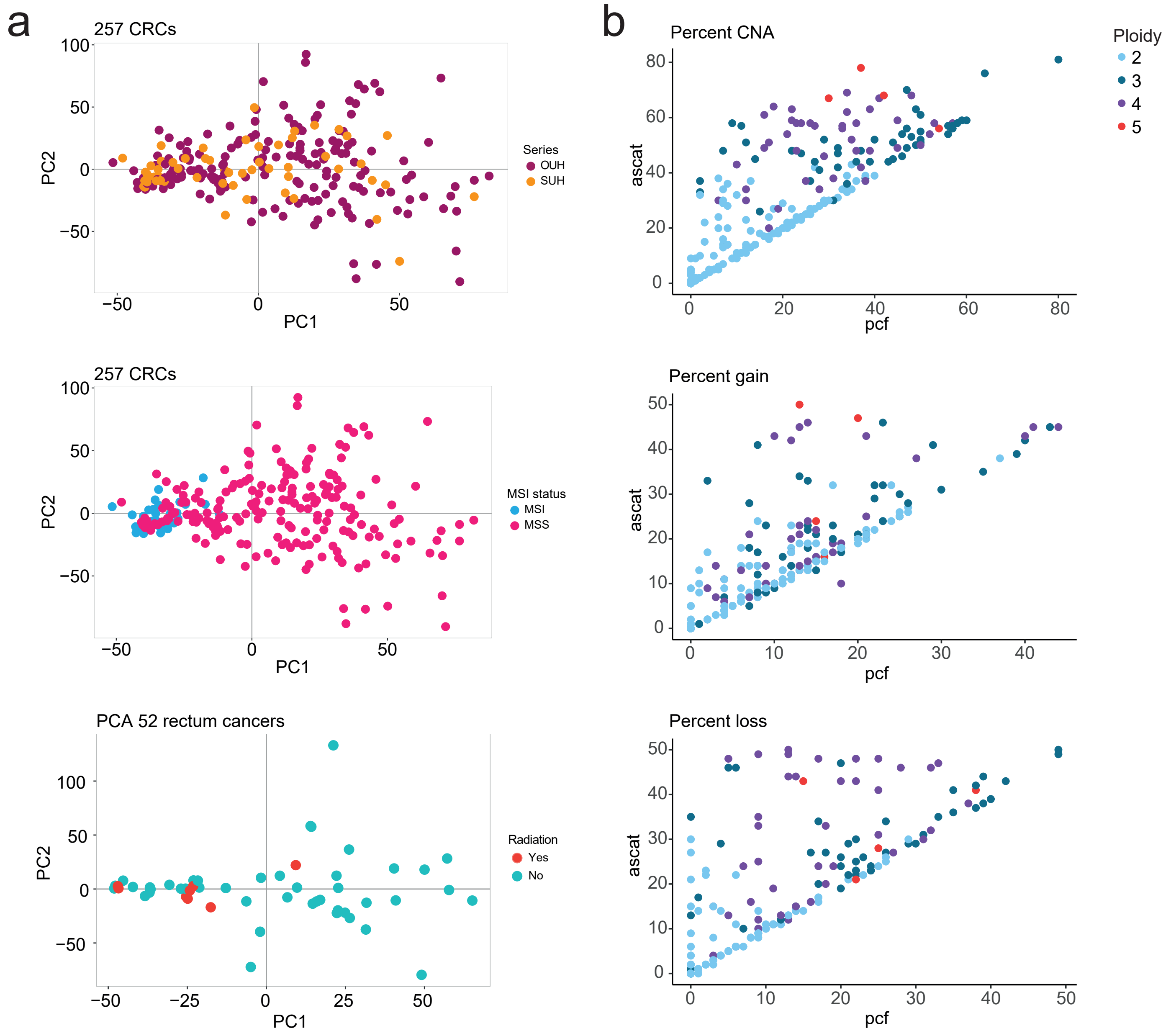

Supplementary Figure 6. a) Principal component analysis of CNA data (decomposed to segments comprising the smallest region of overlap between all samples) showed that the segmented copy number data from tumors collected at two different locations (OUH and SUH) did not differ systematically in DNA copy number (top). MSI tumors clustered together along principal component 1 (PC1; middle), and although most radiated rectum samples clustered in the same region along PC1, they did not form a separate cluster apart from the other rectum tumors and were therefore included in CNA analyses (bottom). b) The estimated percentage of genome with copy number aberrations according to two applied algorithms for CNA data segmentation. Due to the way gain and loss were defined, the ASCAT data produced higher estimations of gain and loss, as the allele-specific data is normalized against the median adjusted copy number by subtraction. Consequently, PCF data was used for calling gain and loss, while ASCAT was used where absolute copies were required for the analysis. The correlation between the percentage of CNAs per sample produced by the two algorithms was high (Spearman rho 0.85).

Supplementary Figure 7

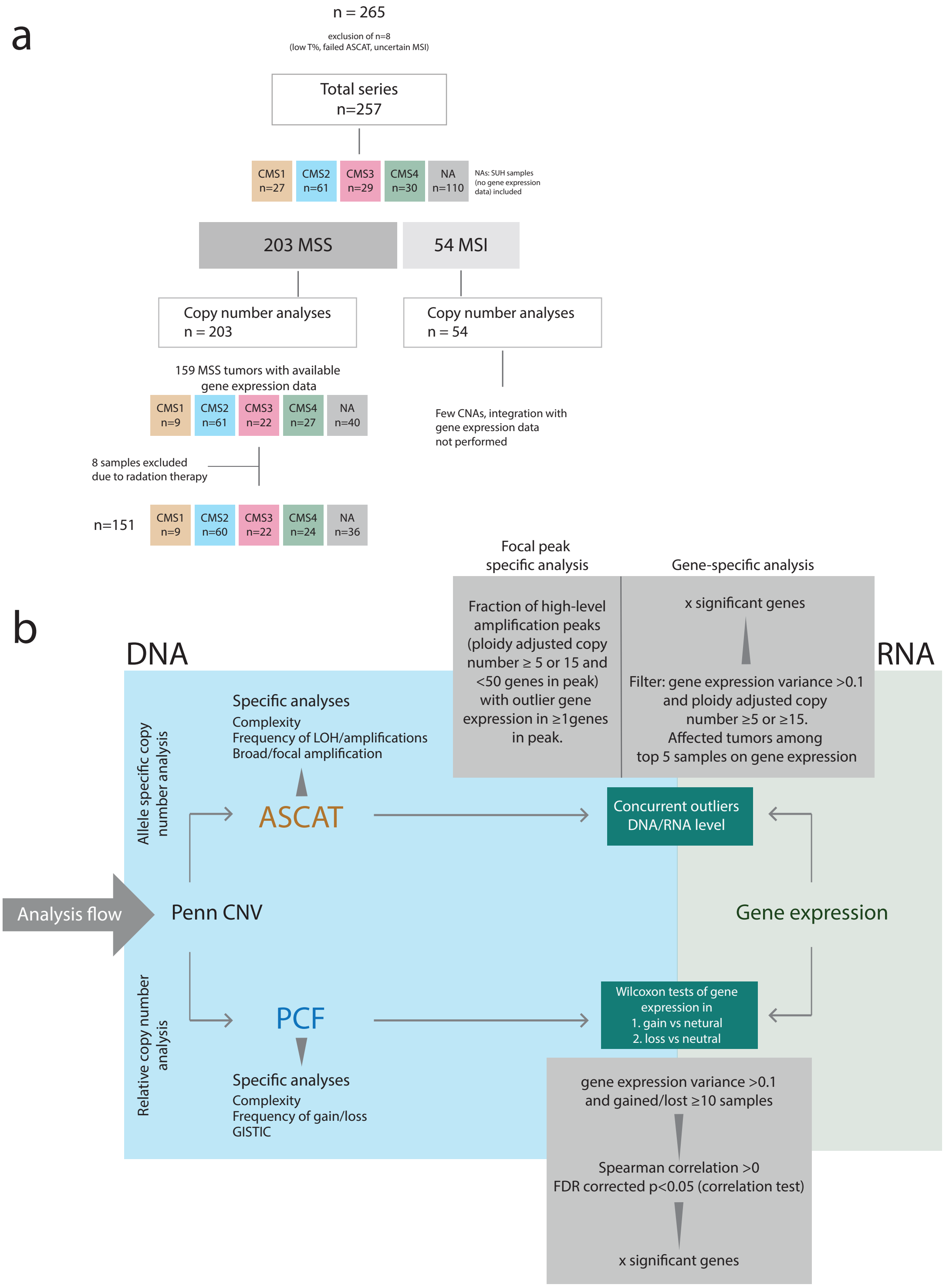

Supplementary Figure 7. a) Overview of material. A total of 265 samples were collected for analyses, and 8 samples were excluded for various reasons prior to downstream bioinformatic analyses. The final series comprised 203 MSS, 53 MSI tumors, and 1 POLE mutated MSS tumor. For 159 MSS tumors from the OUH series, corresponding gene expression data were available, and 151 MSS tumors were included in gene expression related analysis while 8 tumors treated with radiation therapy were excluded. b) Analysis workflow for integrative analyses of DNA/RNA data.
